# Comparison of a personalized breast dosimetry method with standard dosimetry protocols

**DOI:** 10.1038/s41598-019-42144-7

**Published:** 2019-04-10

**Authors:** Elisabeth Salomon, Peter Homolka, Friedrich Semturs, Michael Figl, Michael Gruber, Johann Hummel

**Affiliations:** 1 Medical University of Vienna, Center for Medical Physics and Biomedical Engineering, Vienna, 1090 Austria; 2 Radiologische Gruppenpraxis Baden, Baden, 2500 Austria

## Abstract

Average glandular dose (AGD) in digital mammography crucially depends on the estimation of breast glandularity. In this study we compared three different methods of estimating glandularities according to Wu, Dance and Volpara with respect to resulting AGDs. Exposure data from 3050 patient images, acquired with a GE Senographe Essential constituted the study population of this work. We compared AGD *(1)* according to Dance *et al*. applying custom g, c, and s factors using HVL, breast thickness, patient age and incident air kerma (IAK) from the DICOM headers; *(2)* according to Wu *et al*. as determined by the GE system; and *(3)* AGD derived with the Dance model with personalized c factors using glandularity determined with the Volpara (Volpara Solutions, Wellington, New Zealand) software (Volpare AGD). The ratios of the resulting AGDs were analysed versus parameters influencing dose. The highest deviation between the resulting AGDs was found in the ratio of GE AGD to Volpara AGD for breast thicknesses between 20 and 40 mm (ratio: 0.80). For thicker breasts this ratio is close to one (1 ± 0.02 for breast thicknesses >60 mm). The Dance to Volpara ratio was between 0.86 (breast thickness 20–40 mm) and 0.99 (>80 mm), and Dance/GE AGD was between 1.07 (breast thickness 20–40 mm) and 0.98 (41–60, and >80 mm). Glandularities by Volpara were generally smaller than the one calculated with the Dance method. This effect is most pronounced for small breast thickness and older ages. Taking the considerable divergences between the AGDs from different methods into account, the selection of the method should by done carefully. As the Volpara method provides an analysis of the individual breast tissue, while the Wu and the Dance methods use look up tables and custom parameter sets, the Volpara method might be more appropriate if individual ADG values are sought. For regulatory purposes and comparison with diagnostic reference values, the method to be used needs to be defined exactly and clearly be stated. However, it should be accepted that dose values calculated with standardized models, like AGD and also effective dose, are afflicted with a considerable uncertainty budgets that need to be accounted for in the interpretation of these values.

## Introduction

Breast cancer is the most common cancer in women worldwide and still shows high mortality. While the incidence rate is increasing, the survival rate has improved over the last 20–30 years^1^. This effect is also owed to screening programs which were primarily established in the western hemisphere to decrease mortality. The exposure from mammography poses an additional risk to induce tumors by ionizing radiation. Several papers have examined and estimated the risk arising from such screening programs and compared with the benefit^2–4^.

The dosimetric quantity in mammography best relating to patient dose is Average Glandular Dose (AGD)^5^, which is sometimes also referred to as Mean Glandular Dose (MGD)^6^. Since the AGD cannot be measured directly, conversion factors depending on breast size, breast composition and x-ray spectrum have to be determined. Such conversion factors derived using Monte Carlo simulations on simplified breast models allow to calculate the AGD from measured Incident Air Kerma (IAK)^6^. As shown by Yaffe *et al*.^7^ a simplified breast model considering a composition of 50% adipose and 50% glandular tissue does not represent reality and correction for individual glandularity is necessary.

The most commonly used conversion factors were determined by Dance *et al*.^8^ and were adopted in most beast dosimetry implementations. This formalism was also based on simple breast models and originally provided factors only for cranio-caudal projections and standard Mo x-ray spectra. Further investigations allowed for extensions to other projections as well as for a wide range of x-ray spectra^9^. Conversion factors based on these studies can be found in the EUREF protocols^10,11^ and are used to calculated AGD by

1 $$AGD=IAK\cdot g\cdot c\cdot s$$

*IAK* represents incident air kerma without backscatter, g the conversion factor for a breast with a defined glandularity of 50% by weight. The factor *s* corrects for x-ray spectra if different from Mo/Mo, while *c* corrects for the glandularity of the breast if different from 50%, being 1 in the latter case. Glandularities and c-factors are tabulated as a function of breast thickness (see Table 1) assuming a 0.5 *cm* surface layer of adipose tissue^9^. The factor *g* (as a function of HVL and thickness) describes the fraction of ‘IAK’ that is absorbed by the glandular tissue, assuming 50% glandularity and 50% fat tissue. As described in literature^8,9^ the factor *c* = *c*(*HVL*, *age*, *glandularity*) depends on HVL, age, and glandularity, *g* = *g*(*HVL*, *thickness*) on HVL and thickness, and the factor *s* = *s*(*anode*/*filter*) on the spectrum and is therefore parametrized by the anode/filter combination. Li *et al*.^12^ presented a parametrization method for g and c factors according to Dance *et al*.^9^, which can be used to interpolate g and c factors for arbitrary breast thicknesses and HVLs in the ranges of 2–11 *cm* thickness, and 0.3–0.8 *mm* Al HVL, respectively. As described in the method section this interpolation was used for our calculations. For illustration purpose the g factors for representative breast thicknesses and HVL are also shown in Table 1.

**Table 1:** Glandularity according to Dance for the age group 50 to 64. *c* and *g* factors as result of the parametrization method from Li *et al*.^12^ for different breast thicknesses and HVL.

Thickness	Glandularity	c- factors			g- factors		
		HVL: 0.3 mm Al	0.5 mm Al	0.8 mm Al	0.3 mm Al	0.5 mm Al	0.8 mm Al
20 mm	100	0.863	0.885	0.917	0.390	0.541	0.683
30 mm	72	0.923	0.935	0.952	0.273	0.405	0.555
40 mm	50	0.998	1.00	1.00	0.207	0.319	0.460
50 mm	33	1.08	1.07	1.06	0.165	0.259	0.388
60 mm	21	1.15	1.13	1.11	0.136	0.215	0.333
70 mm	12	1.21	1.19	1.15	0.115	0.182	0.289
80 mm	7	1.26	1.23	1.18	0.099	0.157	0.254
90 mm	4	1.29	1.26	1.21	0.0868	0.138	0.226
100 mm	3	1.31	1.28	1.22	0.0774	0.123	0.204
110 mm	3	1.32	1.29	1.23	0.0699	0.112	0.186

Alternatively, Wu *et al*.^13^ estimated AGD according to

2 $$AGD={X}_{ESE}\cdot DgN$$

where *X*_*ESE*_ represents the breast entrance skin exposure and *DgN* the normalized glandular dose per unit entrance skin exposure. For the calculation of *DgN* equations are provided in^13^ for different anode/filter combinations and different glandularities. Finally, Boone *et al*.^14^ extended Wu’s data with more anode/filter combinations (W/Rh and W/Ag) and higher compressed breast thicknesses.

For individual determination of glandularity, different methods were introduced. Keller *et al*.^15^ presented an algorithm for fully automated quantification of breast percent density based on adaptive multiclass fuzzy c-means clustering and support vector machine classification. Jansen *et al*.^16^ developed a method to determine the glandularity for individual compressed breasts during mammography, based on measurement and calculation of attenuation. There are also commercial solutions available for individual measurement of breast density, such as PowerLook Density Assessment (iCAD Inc., Nashua, NH, USA)^17^, Refine Breast Density Assessment (Philips, Hamburg, Germany)^18^ and Volpara (Volpara Solutions, Wellington, New Zealand)^19^.

The Volpara breast composition determination^20^ uses formula (3) to calculate the thickness of glandular (dense) tissue *h*_*d*_(*x*, *y*) at a given pixel (x, y). Here, *P*_*fat*_ is the pixel value representing adipose tissue, and *μ*_*fat*_ and *μ*_*dense*_ describe the effective linear attenuation coefficients for adipose and glandular (dense) tissue, respectively.

3 $${h}_{d}(x,y)=\frac{{ln}(P(x,y)/{P}_{fat}(x,y))}{{\mu }_{fat}-{\mu }_{dense}}$$

With this approach, it is assumed that the pixel value P(x, y) is linearly related to the energy imparted to the x-ray detector^20^. To determine *P*_*fat*_, the breast boundary in the x-ray image has to be determined by segmentation of the image. For this purpose, a phase congruency model^21^ is used, and an iterative approach with realistic breast edge models^20^ applied to find the fatty breast edge. Breast glandularity is then determined by the ratio of the volume of glandular tissue to total breast volume. While the latter is simply calculated by multiplying the area of the breast by the recorded breast thickness, the volume of the glandular (dense) tissue is computed as the product of *h*_*d*_(*x*) and the pixel area integrated over the entire image. This volumetric breast density (VBD) was converted to glandularity in percentage by weight (GPW) to correspond to Dance’s definition of glandularity. This conversion was done by removing the subcutaneous fat and the uncompressed breast edge from the volume of breast and by changing the density from volume to weight^19^. The breast thickness used bears a particular source of error which was evaluated in the work by Waade *et al*.^22^.

For validation, Lee *et al*.^23^ compared the estimation of the Volpara breast density measurements with the outcome of visual assessment of the BI-RADS breast density category. 860 women were included in this study where experienced radiologists visually assessed mammographic densities using BI-RADS breast density categories^24^. The agreement between these densities and the Volpara densities were evaluated using weighted kappa statistics. Here, a kappa value of 0.799 (95% CI: 0.7708–0.8263) indicating that the Volpara measurements are in good agreement with radiologists’ visual assessment.

Another retrospective study conducted by Rahbar *et al*.^25^ compared the glandularity calculated by the Volpara software with the percent breast density derived from segmented MRI images. Overall, the mean difference between the Volpara estimation and MRI was found to be −0.4%% (95% confidence interval: −1.1 to 0.4%). Nevertheless, at higher breast densities Volpara slightly underestimated glandularities as compared to MRI. In a similar study by Gubern-Merida^26^ also a high correlation between MRI and Volpara glandularity estimation for VBD was found (Pearson correlation coefficients p = 0.93) concluding that accurate volumetric breast density assessment from FFDM images using is feasible and has the potential to be used in personalized risk modeling.

In this paper we compare the AGD using the Dance method^8^ (calculated from imaging parameter found in the DICOM header) and the Wu method^13^ (taken from the DICOM headers) with the AGD calculated by the Volpara software using individual Volpara glandularity measurements.

## Materials and Methods

### Imaging acquisition and population characteristics

**Figure 1:**
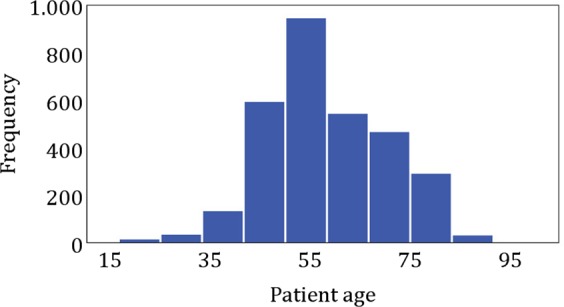
Population characteristics of the examination data used. Histogram of patient age.

3050 anonymized data sets (half of them CC and MLO views, respectively), combined screening and referred women were used for this study. Data on ethnic groups are generally not available in the Austrian health system. All data were acquired with a GE Senographe Essential with software versions ADS_56.10 and ADS_56.21.3, respectively (General Electric, MA, USA). Both versions can be used for 2D mammography and tomosynthesis. For all datasets Volpara version 1.5.2.1 | 7216 | was used, running on a dedicated PC. For all images automatic exposure control (AEC) was employed. Patient age was in the range of 18 to 80 (average 57.9 ± 11.9 years, Fig. 1). Two breast density estimations are provided in the DICOM header, the volumetric breast density (VBD) and the glandularity as percentage by weight (GPW). Dance glandularity (percentage per weight) was calculated from breast thickness and age according to Dance *et al*.^8^.

### Dose calculations

*Volpara AGD* was calculated employing the Dance Eq. (1) using personalized glandularity as GPW (glandularity per weight) and the IAK from the DICOM header. IAK was calculated by the GE software using tabulated tube output per mAs times image mAs corrected for the respective focus skin distance. To determine the *Dance AGD* the parametrization method by Li *et al*.^12^ was employed applying Dance’s *g* and *c*-factors according to Eq. (1).

*GE AGD* (i.e. Wu AGD) provided by the mammographic equipment of GE Healthcare was calculated following the method of Wu *et al*.^13^. The Senographe Essential computes the breast density in the densest part of the image^27^ and the related AGD using normalized glandular dose values interpolated from published data^27^. The normalized glandular dose is defined as the product of the entrance skin exposure, and a conversion factor from exposure to AGD (formula 2)^27^. The AEC performs a pre-exposure image where the densest area of the breast (peak breast density) is determined^27^ and an attenuation-equivalent thickness computed from a calibrated model.

### Statistical analysis

All statistical analysis was done using SPSS (IBM Analytics, NY, USA). GE AGD reported in the DICOM header, calculated Dance AGD and Volpara AGD were analyzed versus compressed breast thickness, HVL, age, glandularity estimation, and entrance air kerma. Linear regression was performed least square, and Pearson correlation factors (*r*) were calculated and given with the p-value of a two-sided test.

## Results

**Figure 2:**
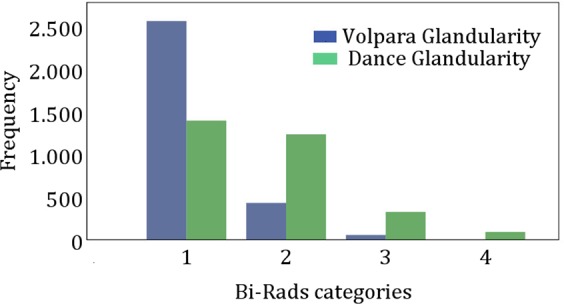
Breast density as a function of BI-RADS categories determined with Dance and Volpara method.

With respect to VBD, most women belonged to BI-RADS category 1 (0% to 25% glandularity), while with respect to the glandularity estimation according the Dance, the number of women were distributed more uniformly between all categories (Fig. 2).

**Figure 3:**
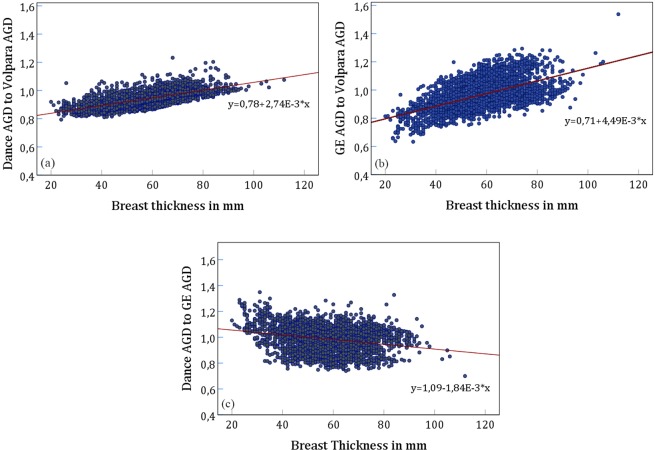
Ratios of the investigated AGDs as a function of breast thickness. (**a**) Dance AGD to Volpara AGD (Pearson *r* = 0.639), (**b**) GE AGD to Volpara AGD (*r* = 0.559), (**c**) Dance AGD to GE AGD (*r* = 0.559), p < 0.001) for all three correlations.

The ratios of GE AGD and Dance AGD to Volpara AGD are shown in Fig. 3 as functions of breast thickness. For small breasts Volpara AGD is higher than GE AGD (Fig. 3(b)). This effect is reversed above a thickness of about 65 mm. The Volpara AGD is also higher than Dance AGD, but the effect is less pronounced and not reversed with higher breast thicknesses (Fig. 3(a)). Figure 3(c) shows the ratio between Dance AGD to GE AGD. Here, Dance AGD is higher than GE AGD for breast thicknesses up to about 60 mm, for thicker breasts GE AGD is higher.

In Table 2 data is pooled into into 4 thickness categories, 20–40 mm, 41–60 mm, 61–80 mm and greater 81 mm. In the smallest thickness group, AGD calculated according to Wu *et al*. (GE AGD) is on average 20% smaller than AGD accounting for individualized density (Volpara AGD). If calculated according to Dance *et al*. with generic values, this difference reduces to 14%. In the thickness categories 41 to 60 mm, and 61 to 80 mm, these differences are at maximum 7%. For the largest breasts (>80 mm) differences between the models are at maximum 2% in the averages. Maximum differences in individual values depending on the method used range from −30% (Dance as compared to GE AGD calculated according to Wu *et al*.) to +48% (between GE AGD and the Volpara AGD) in the largest breasts, and −37 (GE AGD and Volpara) to +35% discrepancy between Dance and GE AGD in the smallest thickness category.

**Table 2:** Ratio of the investigated AGDs split in four breast thickness classes. SD means standard deviation (one σ).

	Breast thickness 20–40 mm					Breast thickness 41–60 mm				
	N	Min	Max	Mean	SD	N	Min	Max	Mean	SD
GE AGD to Volpara AGD	322	0.63	1.05	0.83	0.08	1464	0.74	1.21	0.94	0.09
Dance AGD to Volpara AGD	322	0.79	1.06	0.88	0.04	1464	0.81	1.11	0.92	0.04
Dance AGD to GE AGD	322	0.81	1.35	1.07	0.10	1464	0.75	1.28	0.98	0.10
	**Breast thickness 61**–**80 mm**					**Breast thickness** >**80 mm**				
	**N**	**Min**	**Max**	**Mean**	**SD (1 σ)**	**N**	**Min**	**Max**	**Mean**	**SD (1 σ)**
GE AGD to Volpara AGD	1135	0.81	1.29	1.02	0.10	129	0.85	1.54	1.05	0.10
Dance AGD to Volpara AGD	1135	0.87	1.23	0.97	0.04	129	0.95	1.20	1.02	0.05
Dance AGD to GE AGD	1135	0.74	1.27	0.97	0.10	129	0.70	1.33	0.98	0.09

**Figure 4:**
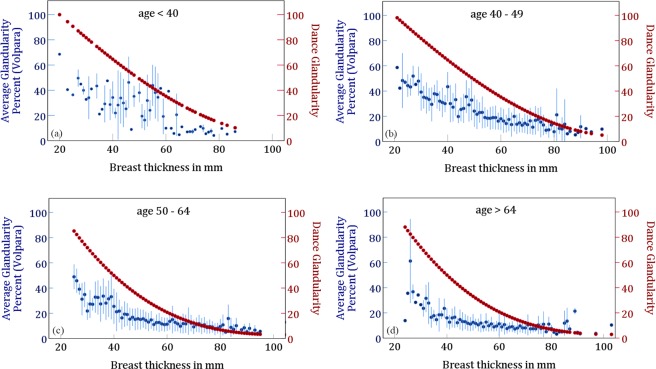
Glandularity depending on breast thickness for 4 different age classes ((**a**) age < 40, (**b**) 40 < age < 49, (**c**) 50 < age < 64, (**d**) age > 64). Red dots: glandularities as defined by Dance *et al*. Blue dots: glandularities calculated by Volpara (whiskers: STD). Below 40 and 65+ years, Dance values from the neighboring categories shown.

In Fig. 4 the glandularity as a function of breast thickness, grouped into four age categories, <40 years, 40–49 years, 50–64 years and >=65 years, is shown. This is important since different glandularity estimations in the AGD calculation methods explain the differences in individual and average breast doses. To consider glandularity as a function of breast thickness and age, multiple linear regression was performed with the Volpara glandularity as dependent, and age and thickness as independent parameters. This results in a linear regression equation of the form *Glandularity* = *c* + *a* * *age* + *b* * *thickness* with *c* = 59.6, *a* = −0.332 and *b* = −0.431. The corresponding ANOVA gives a p-value of <0.001 (*R*^2^ = 0.388), and the following standard errors: 1.048 (c), 0.014 (a), and 0.012 (b), respectively.

Since the Dance glandularity values are only defined for the two middle categories, for patient younger than 40 years the values defined for 40–49 years were taken for comparison, for patient older than 64 years the values defined for the category 50–64 years. While the Volpara values decrease in the first two age categories with breast thickness, above 50 years, the glandularities for breast thickness larger than 40 mm are more or less constant. In contrary, the Dance glandularities decrease in all age categories with the thickness without reaching saturation.

**Figure 5:**
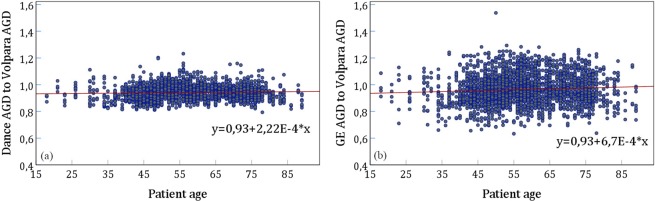
Dose ratios as a function of patient age. (**a**) Ratio of Dance AGD to Volpara AGD (Pearson *r* = 0.065, p < 0.001), (**b**) Ratio of GE AGD to Volpara AGD (Pearson *r* = 0.086, p < 0.001).

**Figure 6:**
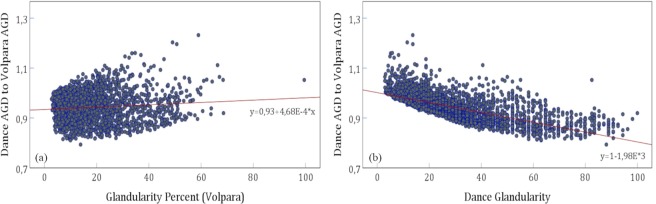
Ratio of Dance AGD to Volpara AGD versus (**a**) Volpara glandularity, (**b**) Dance glandularity. For both correlations the p-value were <0.001.

Figure 5 shows the dependence of the ratios with respect to the patient age. Here, no tendencies can be observed (Pearson coefficient 0.065 and 0.086, respectively). Figure 6 shows the dependence of ratio of AGD calculated according to Dance to AGD calculated with Volpara breast densities on breast glandularity defined either according to Dance *et al*., or calculated by Volpara. For both correlations the p-value were <0.001.

## Discussion

The difference between Dance AGD and Volpara AGD is a direct consequence of the different glandularity estimation models. Whereas the glandularity in the Dance model has a maximum value of close to 100% for very thin compressed breasts (97% for 2 cm thickness), the maximum values according to the Volpara estimation was 68.6%. This difference can also be seen in Fig. 4. The variation between both methods is most pronounced for thin breasts and is more pronounced in older women. Especially in the age groups 50–64 and 65+ (Fig. 4c,d) the effect of involution can be seen in the Volpara glandularities, whereas this effect is not reflected in the Dance approach. However, it needs to be considered that glandularity according to the Dance definition used in this work applies to the central section of the breast surrounded by 5 mm adipose tissue on either side. As the Dance method overestimates the glandularities compared to the Volpara method (see Fig. 4), the resulting c-factors are smaller, and therefore the resulting dose, since the g- and s- factors are the same.

The difference in the glandularities results directly in a difference in dose. Tables 3 and 4 show the resulting dose ratios in percent. This is also illustrated very intuitively if the data is separated into two age categories, age < 50 and age >= 50. Tables 3 and 4 compare the resulting c factors according to the Dance model against the c-factors derived using the Volpara glandularity observed for different compressed breast thicknesses. The HVL taken represents the closest HVL used in our data set to the mean HVLs in the particular thickness range. Differences up to 13 dose ratios (in percent) in the c-values are observed, translating directly into 13% differences in AGDs.

**Table 3:** Differences in c- factors and their expression in dose difference for women younger than 50 years.

Thickness	HVL	*Gland*_*Dance*_	*Gland*_*Volpara*_	*SD*_*Volpara*_	*c*-*value*_*Dance*_	*c*-*value*_*Volpara*_	ratio %
<26	0.380	94.4	46.2	10.8	0.882	1.012	87
[26,39]	0.380	77.4	37	10.9	0.909	1.047	87
[39,50]	0.397	58.3	30.2	13.2	0.967	1.083	89
[50,57]	0.414	44.8	23.1	11.8	1.021	1.121	91
[57,67]	0.430	32.9	17.9	10.6	1.077	1.153	93
[67,83]	0.459	18.6	12.6	8.2	1.157	1.193	97
[>83]	0.459	6.2	9.8	9.4	1.249	1.223	102

**Table 4:** Differences in c- factors and their expression in dose difference for women older than 50 years.

Thickness	HVL	*Gland*_*Dance*_	*Gland*_*Volpara*_	*SD*_*Volpara*_	*c*-*value*_*Dance*_	*c*-*value*_*Volpara*_	ratio %
<26	0.380	91.1	47.4	19.4	0.890	1.009	88
[26,39]	0.380	66.4	28.5	12.5	0.944	1.080	87
[39,50]	0.397	42.9	16.9	9.7	1.028	1.145	90
[50,57]	0.414	29.1	12.4	6.3	1.092	1.177	93
[57,67]	0.430	19.0	10.9	6.5	1.147	1.192	96
[67,83]	0.459	9.2	9.6	6.3	1.214	1.212	100
[>83]	0.459	3.5	9.4	7.4	1.270	1.226	104

The analysis of the ratio of Dance to Volpara AGDs against Dance glandularity (Fig. 6b) shows that smaller ratios reflecting markedly larger AGDs using the Volpara breast density are associated with high glandularity from the Dance model. This effect arises from the large difference between the glandularity values in the range of small breast thicknesses (Fig. 4). For lower Dance glandularity values, the ratio is close to one. This effect cannot be seen for the Volpara glandularity as independent parameter (Fig. 6a).

AGD calculated with the method of Wu by the GE Senograph Essential uses estimated breast glandularity from the densest region of the breast from automatic exposure control, whereas Volpara calculates a whole breast glandularity. The former approaches the total breast glandularity in very dense and extremely fatty breasts, but will be approximately twice as large otherwise^28^. Therefore, the high deviation between GE AGD and Volpara AGD can be better explained by using the glandularity in the densest cm^2^ of the breast for the calculation of the Volpara AGD. In this case the slope found in Fig. 3b decreases from 4.49 to 4.02. However, the effect of glandularity estimation, and other factors influencing *DgN* (Eq. 2) cannot be separated.

Our results of the differences between Dance AGD and GE AGD (see Fig. 3c) are in good agreement with Fedon *et al*.^29^. The difference in skin layer models between Dance and Wu also leads to slightly higher AGD for thin breasts with the Dance model^29^.

Consistency and robustness of the Volpara method has already been demonstrated by Highnam *et al*.^20^. Consistent results were found across four different detectors. Variation of time-current products (mAs) resulted in identical breast densities. Nevertheless, the Volpara approach is limited to breast compositions consisting of only two tissue components (fat and glandular tissue). The occurrence of other tissues such as scar tissue or lesions would result in a systematic deviation of the resulting density. The impact of errors in recorded breast thickness measurements was investigated by Waade *et al*.^22^. The largest change in VBD was found to be 3.1% when applying a 15% error in recorded breast thickness. Another source of error could be the accuracy in the identification of areas containing 100% adipose tissue. Since this pixel value is required for the calculation of the VBD, it has an high impact on the result^20^.

The quality of approximations in Fig. 3 and in the regression model for VBD as a function of age and breast thickness indicates wide variability with both, age and breast thickness. In the work of McCarthy *et al*.^30^ further covariates are used such as body mass index, age at menarche and menopause status. When interpreting the parameters derived from this regression it should also be considered that they only strictly apply to the population in this study. In a different cohort of women, especially with a different ethnicity, they might not apply^30–32^. Therefore, in case personalized values are sought, individual breast glandularity values are necessary.

In general, simplified breast models cannot be used to determine the absolute value of AGD to an individual patient. However, for repeated measurements as for quality control purposes, where relative AGD values are of interest, these models are sufficient^6^.

Breast dosimetry in mammography relies on AGD, as incident dose or kerma, skin dose (kerma) or kerma area product all are inappropriate descriptors of patient dose since their relation to AGD depends on beam characteristics even if the same breast model is used. The concept of AGD relies on computer simulations relating either incident air kerma or entrance skin exposure to dose in glandular tissue. To perform these calculations, a breast model needs to be defined, including distribution of tissues in the breast, glandularity, shape of the compressed breast, to name a few. This model can be defined in varying degrees of sophistication, but never will reflect individual organ shape, composition, tissue distribution in a actual patient. AGD is defined to reflect breast doses to a typical or standard patient, nevertheless assumptions on variation of glandularity with age and compressed breast thickness, must be made. These assumptions will add to uncertainties in both, collective breast doses, as well as individual breast doses.

Depending on which model is used to calculate AGDs, average AGDs in a patient collective will differ by typically up to more than 20%. Therefore, if AGDs are reported, the method needs to be specified. Also, due to the large inter-individual variations in breast composition, it is evident that AGD should not directly be used as individual dose to a patient. However, tomosynthesis might have the potential to allow to calculate more individualized breast doses in future applications.

A limitation of this study is that the analysis is based on clinical data obtained by a single imaging system. Nevertheless, the main statements of this study should be of general validity. Studies based on other systems would be valuable and should be encouraged. It is safe to anticipate these will result in similar AGD ratios but probably different distribution due to a different patient sample.

## Conclusion

As the calculated AGD depends crucially on the glandularity of the particular breast tissue, a reliable determination of this factor is of high importance in dosimetry. We have shown that the choice of the method used to determine glandularity leads to large differences in the resulting AGD. While the methods of Dance *et al*. and Wu *et al*. use glandularities depending on age and breast thickness without taking the particular breast tissue into account, the Volpara method determines glandularity from the individual breast. Therefore, this method paves the way towards a more personalized dosimetry. However, this can be seen as a first step towards real personalized breast dose determination, since other important factors like actual distribution of glandular tissue, eventual presence of lesions that might be classified as healthy glandular tissue, etc, remained unconsidered. Nevertheless, more evaluation studies should be performed to finally prove the validity of this method as described in Lee *et al*.^23^. The magnitude of the uncertainty budget of AGD values is demonstrated by comparing different, well established methods based on real patient data.
